# “I’m Seeing Dead People”: A Case Report on Salicylate Poisoning in a Patient with Hallucinations

**DOI:** 10.5811/cpcem.33609

**Published:** 2025-01-19

**Authors:** Jessica Meyers, Sean McCormick, Phillip D. Levy, Michael J. Twiner

**Affiliations:** * Wayne State University, Department of Emergency Medicine, Detroit, Michigan; † Wayne State University, Integrative Biosciences Center, Detroit, Michigan

**Keywords:** aspirin toxicity, case report, hallucinations, overdose, salicylate poisoning

## Abstract

**Introduction:**

Salicylate poisoning remains one of the most common global accidental overdoses and poses a considerable health threat. Typical presentations for salicylate overdoses include nausea, vomiting, and abdominal pain as well as tinnitus, tachypnea, fever, and dehydration resulting in a concomitant metabolic acidosis and respiratory alkalosis. This may progress to a predominance of neurological symptoms such as mental status changes, confusion, delirium, and hallucinations.

**Case Report:**

We describe the case of an accidental, sub-chronic overdose (up to 7.5 grams/day for multiple weeks; ~75 milligrams/kilogram/day) that resulted in predominantly neurological symptoms (ie, tinnitus and hallucinations, including the patient reporting “seeing dead people”) but without the more typical findings classically associated with salicylate toxicity. The patient was started on a sodium bicarbonate drip; after two days, symptoms completely resolved, and she was safely discharged home.

**Conclusion:**

This case serves as a reminder for physicians to have a high index of suspicion for chronic toxicities including salicylates in patients who present as acute psychosis or altered mental status of unknown etiology.

## INTRODUCTION

Acetylsalicylic acid, or aspirin, is a commonly available over-the-counter nonsteroidal anti-inflammatory drug used to reduce pain, fever, and inflammation. It also inhibits aggregation of platelets, making it cardioprotective. Because it is so readily available and inexpensive, it is often taken at inappropriate doses, which may lead to potentially devastating outcomes, particularly in the elderly. Acetylsalicylic acid continues to rank among the most frequently reported drugs associated with accidental poisonings.^1^ At one point it was estimated that 26% of women aged 65–74 years old were regularly taking aspirin.^2^ Although toxicity and severity of symptoms is related to dose (generally exceeding ingestion of 150 milligrams per kilogram (mg/kg) or serum concentrations of >100 mg/deciliter [dL]), acute poisonings usually manifest as acute nausea, vomiting, and abdominal pain as well as tinnitus, tachypnea, fever, and dizziness.^3^

Subacute and chronic poisonings tend to be quite non-specific and include similar but milder symptoms than acute cases, although progressive confusion, mental status changes, dehydration, and hypotension may be more likely to develop.^3^ Due to non-specific symptoms, chronic toxicity is often misdiagnosed or results in a delayed diagnosis. Furthermore, blood salicylate concentrations in chronic toxicity may be misleadingly lower due to tissue distribution and accumulation in the central nervous system (CNS). This is believed to account for the increased number of neurologic symptoms compared to acute toxicity.^4^ As CNS salicylate concentrations increase, neurologic manifestations such as delirium and hallucinations tend to be more common and profound.^1^ However, there is limited literature describing the neurologic manifestations seen in chronic aspirin toxicity.

In this case report, we present a patient with sub-chronic salicylate toxicity who presented solely with tinnitus and visual hallucinations that resolved once identified and treated.

## CASE REPORT

A 65-year-old female with a medical history significant for hypertension, diabetes, heart failure, and chronic back pain presented to the emergency department by ambulance for visual hallucinations. Without a prior history of any psychiatric disorders or any other complaints, she requested to see a psychiatrist for her visual hallucinations of “I’m seeing dead people.” She even went as far as to discharge a firearm at these “dead people.” Other than elevated blood pressure, her vital signs were within normal limits: blood pressure 148/84 millimeters of mercury; heart rate 87 beats per minute (min); respiratory rate 16 breaths/min; temperature 37.1 degrees Celsius and pulse oximeter 97% on room air. Weight was 100 kg. Physical exam revealed a calm, cooperative patient who was alert and oriented to person, place, and time, and in no acute distress. Physical exam including neurological exam was normal. The patient denied suicidal or homicidal ideations.

Given that the patient had no prior psychiatry history, it was determined she required medical assessment before consulting psychiatry. Initial laboratory studies demonstrated a normal point-of-care glucose at 119 mg/dL. Serum creatinine was slightly elevated at 1.33 mg/dL (reference range 0.6–1.2 mg/dL). She had a microcytic anemia with a hemoglobin of 8.3 grams (g)/dL (11.5–15.1 g/dL) (baseline ≈7–10 g/dL) and mean corpuscular volume of 74 femtoliters (fL) (82–97 fL), white cell count of 9,500 per microliter (μL) (3,500–10,600/μL) and platelet count of 430,000/μL (150,000–450,000/μL) (Table). There were no electrolyte abnormalities, with bicarbonate of 22 millimoles (mmol)/liter (L) (21–31 mmol/L) and anion gap of 10 mmol/L (5–15 mmol/L). Creatine phosphokinase was slightly elevated at 261 micrograms (μg)/L (30–223 μg/L). Urinalysis was negative for an acute urinary tract infection, with a urine pH of 5.5 (5.0–9.0). Urine drug screen was negative.

Influenza, COVID-19, and respiratory syncytial virus swabs were negative. A venous blood gas revealed a mild metabolic acidosis with a pH of 7.33 and a bicarbonate level of 21.6 mmol/L. The treating clinicians also included toxicology screening as part of a broad-spectrum workup, which found acetaminophen, alcohol, and tricyclic levels all below the limit of detection; however, salicylate level was elevated at 28.8 mg/dL. Chest radiograph showed no acute cardiopulmonary process, and non-contrast computed tomography of head showed no acute intracranial process.

At this time, more history was elicited regarding medications with a focus on salicylate use. The patient revealed that she had been taking Bayer^®^ Body and Back pain medication, which contains 500 mg of aspirin and 32.5 mg of caffeine. She admitted to taking upward of 15 pills (7.5 g) a day for multiple weeks, which approximates to 75 mg/kg/day. When explicitly asked, she did endorse tinnitus as well.

**CPC-EM Capsule**

What do we already know about this clinical entity?

*Salicylate poisoning remains one of the most common global accidental overdoses and poses a considerable health threat*.

What makes this presentation of disease reportable?

*This is a case of a sub-chronic overdose of salicylates that presented with hallucinations and tinnitus but no other findings consistent with typical presentations*.

What is the major learning point?

*Physicians must maintain a high index of suspicion for chronic toxicities including salicylates when patients present as acute psychosis of unknown etiology*.

How might this improve emergency medicine practice?

*Physicians should consider a broader toxicological workup when assessing elderly patients with new psychiatric or neurological presentations*.

The patient’s case was discussed with the Michigan Poison and Drug Information Center, which recommended initiation of a sodium bicarbonate drip at 120 mL/hour. This was started, and the patient was admitted to the medicine floor after clearance by the intensivist service. Upon repeat testing 11 hours later, the patient’s salicylate level had decreased to 18.3 mg/dL. Within 48 hours, her visual hallucinations resolved, the bicarbonate drip was discontinued, and she was discharged home on admission day two. Due to resolution of her hallucinations and no concern for self-harm, psychiatry was not consulted.

## DISCUSSION

Aspirin and other salicylate-containing products are over-the-counter medications commonly used and easily available, but when taken inappropriately they can cause toxicity that may be difficult to clinically diagnose. Acute salicylate toxicity classically causes both metabolic acidosis and respiratory alkalosis, tinnitus, and gastrointestinal distress. On the other hand, symptoms and clinical presentations for subacute and chronic poisoning are more subtle with variable manifestations involving multiple organ systems, including a preponderance of neurological symptoms. This case serves as a reminder to emergency physicians that the inappropriate use of aspirin or other salicylate products for multiple weeks (subacute/chronic) can result in potentially subtle (ie, tinnitus) and atypical presentations focused solely on neurological symptoms (ie, visual hallucinations) so extreme that the patient in this case found herself to be psychotic after she used a firearm to shoot at “dead people.” Interestingly, these symptoms were in the absence of the other classical findings of acute or chronic salicylate poisoning.

Salicylates are known to irreversibly inhibit platelet cyclooxygenases, which may be cardioprotective but also increases the risk of bleeding.^5^ In acute poisonings, there is often a rapid loss of potassium through symptomatic vomiting, increased renal excretion of potassium (compensatory response of the initial respiratory alkalosis with increased permeability of the renal tubules), and inhibition of the active transport system.^6^ Severe, acute salicylate poisoning can progress to hyperthermia, coagulopathy, and pulmonary and cerebral edema. Hypoglycemia may develop due to simulation of glucagon release, increased energy demand, depletion of glycogen stores, and decreased gluconeogenesis.^1^ Tinnitus may develop due to activation of cochlear N-methyl-D-aspartate receptor activity, leading to an increase in receptor currents.^7^ Resolution of tinnitus after the exposure generally takes several days.

Subacute and chronic ingestions tend to have more subtle laboratory abnormalities with a predominance of more neurological symptoms. Although the mechanism is poorly understood, longer term ingestions tend to saturate serum albumin, thus allowing free salicylate to more easily cross the blood-brain barrier and potentially contribute to cerebral edema^8^ and other neurological complications. This is particularly more pronounced in elderly patients as they are predisposed to becoming more ill at a lower serum concentration due to decreased hepatic transformation and renal dysfunction, causing reduced elimination/clearance.^8^ Thus, early diagnosis through obtaining a sufficient history with a high clinical suspicion is important, as salicylate poisoned patients may mimic other presentations such as delirium, stroke, sepsis, and psychosis.

Although there is no specific antidote for a salicylate overdose, the mainstay treatment for chronic toxicity is intravenous sodium bicarbonate (as given to our patient), which induces blood and urine alkalinization allowing ionized salicylic acid to more readily exit the body through enhanced renal elimination.^9^,^10^ Upon presentation, our patient’s serum salicylate level was 28.8 mg/dL; after 11 hours on a sodium bicarbonate drip her level had dropped to 18.3 mg/dL. Within 48 hours, the patient was psychosis-free with resolving tinnitus and discharged home.

## CONCLUSION

Patients with chronic salicylate ingestions may present with non-specific neurological complaints (in this case just visual hallucinations and tinnitus) in the absence of the other more-classic symptoms and laboratory abnormalities often seen with salicylates. This case serves as a reminder to clinicians that they should have a high index of suspicion for chronic salicylate toxicity, especially in elderly patients with neurologic or psychiatric presentations.

## Figures and Tables

**Table:** Case patient’s laboratory values.

Laboratory component	Value	Normal range	Units
Serum			
White cell count	9.5	3.5–10.6	1000s/μL
Hemoglobin	8.3	11.5–15.1	g/dL
Mean corpuscular volume	74.2	82–97	fL
Platelets	430	150–450	1000s/μL
Sodium	137	136–145	mMol/L
Potassium	3.8	3.5–5.1	mMol/L
Chloride	105	98–107	mMol/L
Carbon dioxide	22	21–31	mMol/L
Anion gap	10	5.0–15.0	mMol/L
Glucose	108	75–105	mg/dL
Urea nitrogen	17	7.0–25	mg/dL
Creatinine	1.33	0.6–1.2	mg/dL
Calcium	8.8	8.6–10.8	mg/dL
Magnesium	2.2	1.6–3.0	mg/dL
Creatine phosphokinase	261	30–223	units/L
Total protein	8	6.4–8.9	g/dL
Albumin	3.9	3.5–5.7	g/dL
Thyroid stimulating hormone	0.52	0.45–5.33	μIU/mL
Toxicology screens			
Acetaminophen	<10	<25	μg/mL
Salicylates	28.8	<6	mg/dL
Tricyclic acids screen	negative	negative	
Ethanol	<10	<80	mg/dL
Blood gas			
pH	7.332	7.35–7.45	
Partial pressure of carbon dioxide	41.8	36–45	mm Hg
Partial pressure of oxygen	37.6	35–55	mm Hg
Bicarbonate	21.6	22–26	mEq/L
Lactate	1.45	0.6–2.4	g/dL
Nasal			
Viral swabs (COVID-19/influenza/RSV)	negative	negative	
Urine			
Ketones	trace	<3	
Specific gravity	1.029	1.000–1.060	
Blood	negative	negative	
pH	5.5	5.0–9.0	
Nitrite	negative	negative	
Leukocyte esterase	negative	negative	
Urine drug screen	negative	negative	

*dL*, deciliter; *fL*, femtoliter; *g*, gram; *mm Hg*, millimeters of mercury; *mol*, millimole; *mEq*, milliequivalents; *mg*, milligram; *mL*, milliliter; *μIU*, micro international units; *μg*, micrograms; *μL*, microliters; *RSV*, respiratory syncytial virus.
